# Hierarchy hurts: a comparative cross-sectional analysis of empathy and its determinants in medical, midwifery, and nursing students

**DOI:** 10.1186/s12909-025-07683-w

**Published:** 2025-07-18

**Authors:** Lena Maria Weber, Lena Bauer, Amand Führer

**Affiliations:** Institute for Medical Epidemiology, Biometrics and Informatics (IMEBI), Faculty of Medicine, Martin Luther University Halle-Wittenberg, Magdeburger Straße 20, 06112 Halle (Saale), Germany

**Keywords:** Empathy, Elitism, Personality, Medical students, Nursing students, Midwifery students

## Abstract

**Background:**

Empathy plays a crucial role in healthcare, affecting both patient outcomes and provider well-being. Despite its importance, the adaptive nature of empathy remains not fully understood, making it challenging for educational institutions to effectively teach it. This study addresses this gap by investigating two key questions: *What is the relationship between social dominance orientation and empathy among students in medicine*,* nursing*,* and midwifery?* And, *what individual factors – including personality traits*,* sociodemographic background*,* and self-perceived empathy – are associated with variations in empathy across these student groups?*

**Methods:**

This cross-sectional study, conducted in April 2023 at Martin Luther University Halle-Wittenberg, Germany, examined empathy among medical, BSc Midwifery, BSc Nursing, and MSc Health and Nursing Sciences students, aiming to identify its determinants, considering personality traits, socio-economic background, and elitist attitudes. The survey included the Jefferson Scale of Empathy for Health Profession Students (JSE-HPS), NEO-Five-Factor-Inventory (NEO-FFI-30), Narcissistic Personality Inventory (NPI-15), the Short Scale on Social Dominance Orientation (KSDO-3), and the MacArthur Scale. The authors analyzed the data using multiple linear regression models, controlling for relevant confounders.

**Results:**

A total of 252 students participated in the survey, including 173 medical, 35 BSc Midwifery, 24 BSc Nursing, and 20 MSc Health and Nursing Sciences students. Empathy levels varied between disciplines, with BSc Midwifery students exhibiting the highest scores (114.83, 95%-CI = 111.77; 117.88), and BSc Nursing students the lowest (106.25, 95%-CI = 103.1; 109.4). Multiple regression analysis revealed that empathy was positively associated with Openness (β = 2.68, 95%-CI = 1.16; 4.19), Agreeableness (β = 6.01, 95%-CI = 3.9; 8.12), and having children (β = 5.02, 95%-CI = 0.25; 9.79). Conversely, students with non-medical training prior to university (β = -9.86, 95%-CI = -16.84; -2.88) and higher Social Dominance Orientation (β = -0.64, 95%-CI = -1.11; -0.16) showed lower empathy scores.

**Conclusions:**

In addition to confirming previous findings, this study is, to the best of our knowledge, the first to investigate Social Dominance Orientation in the context of healthcare students’ empathy and describe its negative association. The findings suggest that addressing hierarchical attitudes in empathy education could positively impact healthcare delivery.

**Trial registration:**

This was not undertaken as the research did not involve a health care intervention on human participants.

**Supplementary Information:**

The online version contains supplementary material available at 10.1186/s12909-025-07683-w.

## Background

Empathy in patient care has been defined in many ways. In this study, we adopt the definition by Hojat et al. as a “predominantly cognitive attribute” involving understanding patients’ experiences, concerns, and perspectives, communicating this understanding, and having a desire to help [1]. This *Cognitive Empathy* focuses on “reasoning” and “appraisal” [2] and is a conscious act influenced by personal background, learning, and educational experiences [1].

Empathy significantly impacts healthcare delivery, enhancing physicians’ clinical competence [3] diagnostic accuracy [4, 5] patients’ adherence [6] health literacy [7] quality of life [8] and a broad spectrum of clinical outcomes [9] while also reducing healthcare providers’ burnout [10] and malpractice claims [11]. In midwifery, it enhances women’s birth experience [12] and in nursing, it increases patient satisfaction [13] and wellbeing [14]. Consequently, empathy is considered a core skill for healthcare providers, with growing recognition in recent years [15]. However, many studies reveal that empathy tends to decrease as healthcare students advance in their training [16–19].

Empathy, as a cognitive and “intellectual attribute” [20] is an adaptive and teachable skill, and increasingly integrated into medical, nursing, and midwifery curricula worldwide [21, 22]. To enhance these programs’ effectiveness, it is necessary to further clarify the parameters affecting empathy in healthcare students.

The literature points to several variables explaining the decline of healthcare students’ empathy throughout their studies, including high workload [23, 24] stress [25, 26] the hidden curriculum [16] negative role-modeling [22, 27] and medical authoritarianism [28, 29]. On an individual level, personality traits [30, 31] the psychosocial background [28, 29, 32] the feeling of need for self-preservation [5, 16, 23] and spiritual well-being [29] feature most prominently as determinants of empathy.

### Doctor-patient relationship

The relationship between patient and healthcare provider is inevitably shaped by power asymmetries, which may complicate empathetic engagement. Yet, a preference for hierarchies and inequality is rarely considered in analyses of empathy within the medical field, even though it may meaningfully influence how empathy is valued and expressed in clinical education and practice. Historically, healthcare providers, particularly physicians, held substantial authority due to their exclusive medical expertise. Drawing from Weber’s concept of authority, this translated into both traditional authority, rooted in long-established practices, and charismatic authority, stemming from their specialized skills and the ability to inspire trust and confidence in patients [33, 34]. In recent years, healthcare education has increasingly promoted models of shared decision-making, aiming to reduce hierarchical dynamics and foster greater patient involvement, while also empowering nurses and midwives [35]. However, despite these shifts, hierarchical attitudes may persist among healthcare students. There is limited research exploring how such attitudes might shape students’ empathy and, consequently, healthcare delivery.

### Social dominance orientation

To better understand these hierarchical perspectives, we turn to the concept of Social Dominance Orientation (SDO)– a construct that captures an individual’s preference for group-based hierarchies and social inequality. SDO has consistently been shown to be inversely correlated with empathy and Agreeableness in the general population [36–38]. Hereby, empathetic and agreeable individuals are less inclined towards dominance and more concerned with others’ welfare. Those with low Agreeableness, in turn, are more self-interested and “tough-minded” [39]viewing the world as a competitive “Darwinist jungle in which might is right and winning is everything” [39] and where the way to success is through power and dominance [36, 39–42].

Previous studies examining SDO and empathy in the general population have primarily utilized general psychological frameworks to measure empathy (e.g., Interpersonal Reactivity Index [43]or the Compassion facet of Agreeableness [44]). The only study that examined the relationship between SDO and empathy using the Jefferson Scale of Empathy (JSE) as a measure for professional empathy focused exclusively on first-year medical students with limited clinical experience [28]thus providing limited insight into clinical settings. Therefore, this study addresses these limitations by using the JSE to assess empathy among a diverse cohort of healthcare students, including medical, nursing, and midwifery students, with substantial clinical exposure. By bringing SDO into the clinical context, this study contributed to a more nuanced understanding and offers relevant implications for healthcare training and practice.

### Empathetic care as a collaborative effort

While empathetic care is a shared, interprofessional responsibility [45] it is often studied within individual professions, overlooking common challenges and opportunities. Despite different career paths, medical, nursing, and midwifery students share educational resources and faculty. This study recognizes patient care as a collaborative effort across disciplines, providing a more accurate representation of the educational environment. Unfortunately, few studies focus on these issues, particularly concerning nursing and midwifery students [16, 46].

### Research question

This study explores the determinants of empathy among healthcare students across the disciplines of medicine, nursing, and midwifery at a single institution. Specifically, it investigates how hierarchical attitudes, measured through Social Dominance Orientation, relate to levels of professional empathy, a relationship that is largely unexplored in healthcare education. By also incorporating individual factors, this study aims to provide a more nuanced understanding of how empathy manifests and varies among future healthcare providers.

Accordingly, the research is guided by the following questions:

1. What is the relationship between social dominance orientation and empathy among students in medicine, nursing, and midwifery?
2. What individual factors – including personality traits, sociodemographic background, and self-perceived empathy – are associated with variations in empathy across these student groups?

These questions aim to inform the ongoing development of empathy training in healthcare curricula by identifying key social and psychological variables that may contribute to its variations or decline during professional development.

## Methods

We conducted a cross-sectional survey among students of different health professions at the Martin Luther University Halle-Wittenberg, Germany, in April 2023. In the German higher education system, undergraduate and graduate training in health professions is divided into profession-specific tracks with varied clinical exposure. Medical education follows a six-year curriculum, including a two-year preclinical phase, a three-year clinical phase, and a final practical year working full time in healthcare delivery. We included medical students in their fourth to sixth year to ensure clinical experience and prior active engagement in patient care.

The BSc Nursing and Midwifery programs are both structured as four-year degrees introduced in alignment with the European Bologna Process. These programs integrate clinical experience from the first year onward through structured placements in hospital and community settings. Traditionally, both nursing and midwifery in Germany were trained through vocational education, and academization is a relatively recent development. Midwifery has fully transitioned to a university-based degree in 2020, whereas nursing continues to exist in parallel tracks: it can be completed either through vocational training or through an academic program. The midwifery program at our institution was introduced only in the winter semester of 2021/2022, so at the time of the study, only first- and second-year students were enrolled.

The MSc in Health and Nursing Sciences is a two-year interdisciplinary graduate program focused on academic and research skills for healthcare professionals. Although not primarily clinically oriented, most students have prior clinical training and experience.

Empathy education in Germany is increasingly recognized across healthcare curricula, though its implementation remains uneven. While some medical programs include formal instruction in communication and empathy, such training is often less explicitly structured in midwifery and nursing education, where these skills are more commonly developed through experiential learning in clinical settings. To ensure a meaningful comparison, all students had clinical exposure through placements or prior training.

### Questionnaire

The questionnaire was in German and included four main components:

I) **Empathy**, measured using the Jefferson Scale of Empathy for Health Profession Students, developed by Hojat and his colleagues based on their definition of empathy in patient care, and chosen for its validity and reliability [17, 47]. JSE-HPS empathy scores can be further divided into the subscales Perspective Taking (PT), Compassionate Care (CC), and Walking in Patient’s Shoes (WPS). In addition, participants were asked to self-rate their empathy on a single-item Likert scale from 1 (not at all empathetic) to 10 (completely empathetic). This subjective empathy score served as a self-assessment tool to explore how students perceive their empathy and was later contrasted with the validated instrument.
II) **Personality**, assessed with the NEO-Five-Factor-Inventory (NEO-FFI-30), capturing expressions of the five robust personality factors– Neuroticism, Extraversion, Openness to Experience (further only mentioned as Openness), Agreeableness, and Conscientiousness [48] and the Short Version of the Narcissistic Personality Inventory (NPI-15), assessing subclinical narcissistic tendencies [49, 50];
III) **Elitist attitudes**, measured by the Short Scale on Social Dominance Orientation (KSDO-3), assessing a preference for social inequality and hierarchical societies [36, 41, 51].
IV) **Socio-demographic questionnaire**, which was developed by the authors and included questions on the respondents’ personal and socio-economic background, as well as the German version of Nancy Adler’s MacArthur Scale of Subjective Social Status [52].

See Table 1 for more details on the instruments; an English-language version of the socio-demographic questionnaire can be found in Appendix 1.

For completeness, the questionnaire also included an instrument on positional behavior and thinking [53]. However, due to insufficient responses, these data were excluded from analysis and will not be discussed further.

The survey was conducted using an online questionnaire (implemented in Limesurvey) and pretested with students outside of the test population.

**Table 1:** Structure of the questionnaire and instruments used to assess empathy, personality, elitist attitudes, and socio-demographic profile in healthcare students

Instrument	Purpose	Description	Scale	Reference
Jefferson Scale of Empathy-Health Profession Student Version (JSE-HPS)	Assess empathy in patient care	20 statements addressing the role of healthcare providers and their relationship to patients. Includes subscales: Perspective Taking (PT), Compassionate Care (CC), Walking in Patient’s Shoes (WPS)	7-point Likert scale; Total score range: 20–140	Hojat et al. 2004; Preusche et al. 2013
Self-evaluation of empathy	Assess perceived empathy	Single-item Likert scale from 1 (not at all empathetic) to 10 (completely empathetic)	10-point Likert scale; Total score range: 1–10	n.a.
NEO-Five-Factor-Inventory (NEO-FFI-30)	Assess personality traits	30 items capturing the five robust personality factors: Neuroticism, Extraversion, Openness to Experience, Agreeableness, Conscientiousness.	5-point Likert scale; Total score range for each personality trait: 0–4	Costa & McCrae 1989; Körner, Geyer & Roth et al. 2008
Narcissistic Personality Inventory (NPI-15)	Assess subclinical narcissism	15 forced-choice items each offering one narcissistic and one non-narcissistic option	Forced-choice; Total score range: 0–15	Raskin et al. 1988; Spangenberg et al. 2013
Short Scale on Social Dominance Orientation (KSDO-3)	Assess social dominance orientation	3-item scale measuring preference for social inequality and hierarchical societies	5-point Likert scale; Total score range: 3–15	Ho et al. 2015; Aichholzer 2019
Socio-Demographic Questionnaire	Collect socio-demographic data	Includes questions on age, gender, place of origin, faith, family composition, grades, training, parental professions, part-time employment, income and experience with illness	n.a.	Developed by the authors
MacArthur Scale of Subjective Social Status	Assess subjective social status (SSS)	Respondents were asked to place themselves on the “social ladder”	Range: 1–10	Hoebel et al. 2015

### Sampling

565 students from four health profession programs at the Martin Luther University Halle-Wittenberg, Germany, were invited through compulsory classes, where the study was briefly introduced, and then students were given time to complete the questionnaire, if they wanted. In addition, the survey was advertised in online messenger groups of the corresponding courses and student e-mail newsletters. Participation was voluntary and anonymous, with reminders sent after two and three weeks. Recruitment of participants took place in April 2023.

### Statistical analysis

We performed analysis in three steps: (I) descriptive analysis, (II) stratified analysis, and (III) multiple linear regression:

I) In **descriptive analysis**, we report absolute and relative frequencies, means, and 95%-confidence intervals for key variables. To facilitate a direct comparison between students’ self-assessed empathy and their scores on the validated Jefferson Scale of Empathy, we applied a min-max transformation to rescale the original 20 to 140 range of the JSE-HPS scores to the 1 to 10 range used by the single-item self-assessment on empathy, using the following formula:

$$\:Rescaled\:Score=1+\left(\frac{JSE\:Score-20}{120}\times\:9\right)$$

This transformation ensured a proportional alignment between the two measures, allowing an exploratory comparison between subjective and standardized empathy ratings.

We also adjusted the SDO sum score to a mean score to align with the 16-item version used in other studies.

II) In **stratified analyses**, we group participants by personality, social dominance orientation, and socio-demographic factors (e.g., age, gender, study subject) to compare these groups’ mean overall empathy, PT, CC, and WPS.
III) In **multiple linear regression**, we include variables identified as conclusive through stratified analysis, as well as previous research and theoretical considerations. Specifically, we include age and gender, as they are frequent confounders and factors known to influence empathy [29] and neuroticism [29], as it is known to affect emotional responses and interpersonal interactions [30].

All analyses were conducted using RStudio (2021.09.0 + 351 “Ghost Orchid” Release for macOS).

## Results

252 students participated in the survey, including 173 medical (4th– 6th year), 35 BSc Midwifery, 24 BSc Nursing, and 20 MSc Health and Nursing Sciences students. This represents a response of 42%, 100%, 60%, and 67%, respectively.

The sample’s sociodemography, empathy scores, and SDO results are shown in Tables 2 and 3. For more details on the participants’ sociodemographic background and personality traits, see Appendices 2 and 3.

**Table 2:** Demographic characteristics of the study population by study program (1/2)

		Medicine		BSc Midwifery		BSc Nursing		MSc HNS	
		*N* = 173		*N* = 35		*N* = 24		*N* = 20	
		*n*	%	*n*	%	*n*	%	*n*	%
Gender	Female	132	76.3%	34	97.1%	17	70.8%	18	90%
	Male	38	21.9%	-	-	6	25%	2	10%
	Diverse	1	0.6%	-	-	1	4.2%	-	-
	Missing	2	1.2%	1	2.9%	-	-	-	-
Age [years]	Median, range	24	21–40	22	20–39	22	20–37	28.5	24–52
	Missing	1	0.6%	1	2.9%	-	-	-	-
Children	No	165	95.4%	28	80%	21	87.5%	15	75%
	Yes	8	4.6%	5	14.3%	3	12.5%	5	25%
	Missing	-	-	2	5.7%	-	-	-	-
Year of study	Median, range	4	4–6	1	1–2	3	1–3	1	1–3
	Missing	-	-	1	2.9%	-	-	-	-
Training prior to university studies	None	142	82.1%	27	77.1%	23	95.8%	1	5%
	Medical	21	12.1%	6	17.1%	1	4.2%	19	95%
	Non-medical	6	3.5%	1	2.9%	-	-	-	-
	Missing	4	2.3%	1	2.9%	-	-	-	-
Subjective social status	Median, range	7	2–10	5	2–8	5	2–7	6	3–8
	Missing	2	1.2%	1	2.9%	-	-	-	-
Faith	No	113	65.3%	22	62.9%	19	79.2%	12	60%
	Yes	60	34.7%	12	34.3%	5	20.8%	8	40%
	Missing	-	-	1	2.9%	-	-	-	-

Abbreviations. MSc HNS = MSc Health and Nursing Sciences

**Table 3:** Empathy and social dominance orientation of students by study program

		Medicine		BSc Midwifery		BSc Nursing		MSc HNS	
		*N* = 173		*N* = 35		*N* = 24		*N* = 20	
	range	mean	95%-CI	mean	95%-CI	mean	95%-CI	mean	95%-CI
Empathy, SA	1–10	7.76	7.59; 7.93	8.43	8.2; 8.66	8.25	7.82; 8.68	8.10	7.65; 8.55
JSE-HPS									
Total	20–140	111.82	110.34; 113.29	114.83	111.77; 117.89	106.25	103.01; 109.4	111.05	106.7; 115.4
PT	10–70	58.10	57.26; 58.95	61.49	59.82; 63.15	57.50	55.86; 59.14	58.75	56.03; 61.47
CC	8–56	45.48	44.71; 46.25	44.80	43.10; 46.5	40.13	38.03; 42.22	43.75	41.61; 45.9
WPS	2–14	8.23	7.88; 8.58	8.54	7.88; 9.20	8.63	7.81; 9.38	8.55	7.6; 9.5
SDO	3–15	6.88	6.50; 7.27	6.46	5.62; 7.29	7.67	6.7; 8.64	6.60	5.59; 7.61

Abbreviations. MSc HNS = MSc Health and Nursing Sciences, SA = Self-assessment, JSE-HPS = Jefferson Scale of Empathy for Health Profession Students, PT = Perspective Taking, CC = Compassionate Care, WPS = Walking in Patient’s Shoes, SDO = Social Dominance Orientation

### Empathy levels among different student groups

The overall mean JSE-HPS score was 111.64 (95%-CI: 110.43; 112.86). BSc Midwifery students showed the highest empathy scores, followed by medical students and MSc Health and Nursing Sciences students. BSc Nursing students scored the lowest.

To complement the standardized measure, participants were also asked to self-assess their empathy on a 10-point scale, allowing an exploratory comparison between subjective and measured empathy. Interestingly, the subjective self-ranking differed slightly: BSc Midwifery students again reported the highest values, followed by BSc Nursing students, MSc Health and Nursing Sciences students, and finally medical students.

To facilitate comparison, JSE-HPS scores were rescaled to a 1–10 range using linear min-max transformation. This revealed that medical students were the only group to underestimate their empathy levels, with their self-assessment (SA) being closest to their JSE-HPS score (SA = 7.76; JSE-HPS = 7.89; Δ = +0.13), whereas nursing students showed the greatest overestimation (SA = 8.25; JSE-HPS = 7.47; Δ = -0.78). Midwifery students (SA = 8.43, JSE-HPS = 8.11; Δ = -0.32) and health and nursing sciences students (SA = 8.1, JSE-HPS = 7.83; Δ = -0.27) also overestimate their empathy, though to a lesser extent.

### Factors influencing empathy

Our exploratory stratified analyses revealed the following associations with empathy among healthcare students:

A) **Devotion**: Spiritual or religious students scored higher on the JSE-HPS compared to their non-religious peers, with similar trends in the CC subscale.
B) **Social status**: Higher subjective social status (SSS) (MacArthur score of 6–10 (out of 10)) was associated with increased empathy compared to lower SSS (MacArthur score = 1–5 (out of 10)), also in the CC subscale.
C) **Siblings**: Being the oldest sibling was positively associated with empathy levels compared to being the youngest sibling, similarly in the subcategory PT.
D) **Parenthood**: Having children had a positive association with higher empathy levels, but even more so in PT (mean = 61.76, 95%-CI: 59.73; 63.79) compared to not having any children (mean = 58.22, 95%-CI: 57.5; 58.94).
E) **Vocational training**: Non-medical vocational training prior to university had a negative association with empathy compared to having medical training or no training at all. Similar differences were also observed in the subscale WPS.
F) **Personality**: High levels of Agreeableness *(*β = 6.29, 95%-CI: 4.50; 8.08) and Openness *(*β = 2.62, 95%-CI: 1.04; 4.20) were both positively associated with higher overall empathy and all its subdimensions, PT, CC, and WPS.
G) **Social Dominance Orientation**: Higher SDO scores were negatively associated with total empathy scores *(*β = -1.12, 95%-CI: -1.59; -0.65), PT, and CC.

**Fig. 1:**
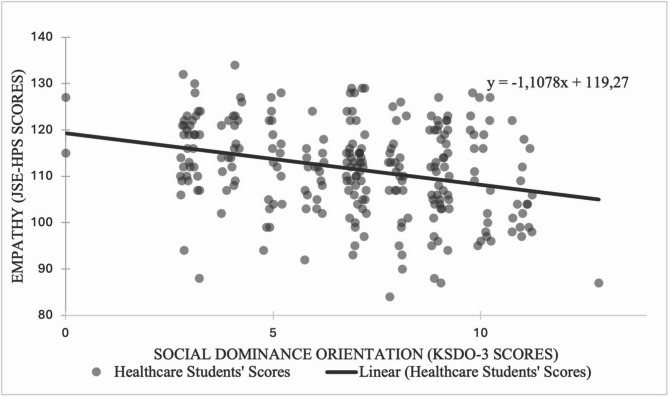
Relationship between Social Dominance Orientation and Empathy among Healthcare Students. Figure 1. Scatter plot showing the relationship between Social Dominance Orientation (SDO) and Empathy (JSE-HPS) among healthcare students. The data was collected from 252 healthcare students from Martin-Luther-University Halle-Wittenberg in April 2023. Each dot represents the SDO score plotted against the corresponding empathy score for an individual student. A slight negative linear relationship is observed, as indicated by the trendline (y = -1.1078x + 119.27). The results suggest that as Social Dominance Orientation increases, empathy scores tend to decrease slightly among this sample of healthcare students

For more details on the JSE-HPS empathy scores stratified by individual factors, refer to Tables 4 and 5; Fig. 1.

**Table 4:** Mean JSE-HPS scores stratified by study program and socio-economic profile

		*n*	Mean	95%-CI	SD
Study program	BSc Midwifery	35	114.83	111.77; 117.89	9.22
	Medicine	173	111.82	110.34; 113.29	9.92
	MSc HNS	20	111.05	106.70; 115.40	9.93
	BSc Nursing	24	106.25	103.10; 109.40	7.88
Gender	Diverse	2	112.50	111.52; 113.48	0.71
	Female	201	112.04	110.73; 113.35	9.47
	Male	46	109.85	106.56; 113.14	11.39
Position in order of siblings	Oldest	101	112.97	111.11; 114.84	9.57
	Middle	31	110.84	107.47; 114.21	9.57
	Youngest	80	110.04	107.92; 112.15	9.65
Children	Yes	21	115.48	111.40; 119.55	9.53
	No	229	111.21	109.94; 112.48	9.77
Training prior to university studies	Medical	47	112.89	109.90; 115.89	10.47
	None	193	111.52	110.17: 112.87	9.57
	Non-medical	7	103.14	95.19; 111.09	10.73
Subjective social status	High (MacArthur = 6–10)	152	112.61	111.06; 114.15	9.74
	Low (MacArthur = 1–5)	97	110.07	108.10; 112.05	9.92
Faith	Yes	85	113.71	111.67; 115.74	9.56
	No	166	110.57	109.07; 112.06	9.83

Abbreviations. JSE-HPS = Jefferson Scale on Empathy for Health Profession Students, MSc HNS = MSc Health and Nursing Sciences

**Table 5:** Mean JSE-HPS scores stratified by personality traits and elitist attitudes

Personality trait		mean	95%-CI	SD
Extraversion^a^	0–1 (*n* = 2)	100.50	74.04; 126.96	19.09
	1.01–2 (*n* = 82)	110.38	108.44; 112.32	8.96
	2.01–3 (*n* = 140)	112.16	110.55; 113.77	9.70
	3.01–4 (*n* = 28)	113.57	109.24; 117.90	11.70
Agreeableness^b^	0–1 (*n* = 4)	93.75	89.27; 98.23	4.57
	1.01–2 (*n* = 19)	106.37	102.26; 110.48	9.15
	2.01–3 (*n* = 84)	109.41	107.43; 111.38	9.23
	3.01–4 (*n* = 145)	114.12	112.61; 115.64	9.28
Conscientiousness^c^	0–1 (*n* = 2)	98.50	75.96; 121.04	16.26
	1.01–2 (*n* = 10)	106.80	99.10; 114.50	12.43
	2.01–3 (*n* = 109)	111.28	109.44; 113.11	9.75
	3.01–4 (*n* = 131)	112.52	110.91; 114.13	9.42
Neuroticism^d^	0–1 (*n* = 66)	111.62	109.21; 114.03	10.00
	1.01–2 (*n* = 101)	111.39	109.57; 113.20	9.30
	2.01–3 (*n* = 72)	112.63	110.25; 115.00	10.08
	3.01–4 (*n* = 13)	108.31	102.66; 113.96	10.40
Openness^e^	0–1 (*n* = 6)	109.83	98.92; 120.75	13.64
	1.01–2 (*n* = 46)	109.07	106.10; 112.04	10.28
	2.01–3 (*n* = 117)	111.44	109.80; 113.09	9.06
	3.01–4 (*n* = 83)	113.48	111.31; 115.65	10.10
NPI-15^f^	0–4 (*n* = 172)	112.13	110.66; 113.60	9.86
	5–9 (*n* = 67	110.84	108.55; 113.12	9.56
	10–14 (*n* = 13)	109.39	103.54; 115.23	10.75
**Tendency towards elitist attitudes**		**mean**	**95%-CI**	**SD**
SDO^g^	3–6 (*n* = 96)	114.24	112.44; 116.04	8.98
	7–10 (*n* = 139)	110.73	109.07; 112.38	9.95
	11–15 (*n* = 17)	104.47	100.31; 108.63	8.75

Abbreviations. NPI-15 = Narcissistic Personality Index, KSDO-3 = Short Scale on Social Dominance Orientation

(a) high Extraversion/low Extraversion = outgoing/reserved. (b) high Agreeableness/low Agreeableness = friendly/self-interested. (c) high Conscientiousness/low Conscientiousness = organized/spontaneous. (d) high Neuroticism/low Neuroticism = nervous/resilient. (e) high Openness/low Openness = curious/cautious. (f) higher NPI-15 scores indicate higher subclinical narcissism. (g) higher SDO scores indicate a stronger preference for social hierarchies

### Regression analyses

Our multivariable regression analyses found that the following factors were associated with empathy levels among healthcare students:

1. **Agreeableness** showed a strong positive association with overall empathy, and all its subcategories, including CC (β = 2.69, 95%-CI: 1.51; 3.86), PT (β *=* 2.59, 95%-CI: 1.34; 3.84), WPS (β = 0.73, 95%-CI: 0.19; 1.27).
2. **Openness** was also positively associated with empathy, particularly with CC (β = 1.66, 95%-CI: 0.81; 2.5) and PT (β = 1.42, 95%-CI: 0.52; 2.31).
3. **Having children** was also positively associated with empathy, particularly PT (β = 3.69, 95%-CI: 0.86; 6.52).
4. **Social Dominance Orientation** was negatively associated with empathy, particularly PT (β = -0.35, 95%-CI: -0.63; 0.07).
5. **Previous non-medical training** was linked to lower empathy, specifically WPS (β = -2.57, 95%-CI: -4.36; -0.77).
6. **Medical studies** and **midwifery studies** showed positive associations with empathy.

Table 6 provides detailed results of the multiple linear regression models for the total JSE-HPS score and Appendix 4 on its subscales CC, PT, and WPS.

**Table 6:** Multiple linear regression model for the outcome total JSE-HPS scores

	Coefficient	95%-CI
Age [years]	-0.29	-0.66; 0.08
Agreeableness	6.01	3.90; 8.12
Conscientiousness	2.19	-0.11; 4.49
Extraversion	0.27	-1.85; 2.38
Having Faith	0.88	-1.46; 3.22
Having children	5.02	0.25; 9.79
MSc HNS studies (reference: BSc Nursing studies)	0.67	-5.42; 6.76
SDO	-0.64	-1.11; -0.16
Male gender (reference: female gender)	-0.41	-3.43; 2.60
Subjective social status	0.34	-0.33; 1.00
Medical studies (reference: BSc Nursing studies)	4.79	0.95; 8.63
BSc Midwifery studies (reference: BSc Nursing studies)	5.26	0.61; 9.91
Neuroticism	1.22	-0.28; 2.72
Non-medical training (reference: medical training)	-9.86	-16.84; -2.88
No training (reference: medical training)	-3.64	-7.75; 0.48
NPI	0.31	-0.15; 0.76
Openness	2.68	1.16; 4.19

Multiple R-squared: 0.34 Adjusted R-squared: 0.29

F-statistic: 6.96 on 17 and 226 DF *p*-value: 1.91e^− 13^

Abbreviations. MSc HNS = MSc Health and Nursing Sciences, SDO = Social Dominance Orientation, NPI = Narcissistic Personality Index

## Discussion

This study examined the relationship between empathy and social dominance orientation among medical, BSc Midwifery, BSc Nursing, and MSc Health and Nursing Sciences students, as well as how individual-level characteristics, such as personality traits and sociodemographic background, may shape empathy across these student groups.

Our central finding is a negative association between SDO and empathy, suggesting that stronger hierarchical orientation may conflict with empathetic professional engagement and thus influence interpersonal behavior and attitudes relevant to patient care and interprofessional collaboration. Additional findings suggest that variations in empathy levels across student groups can also be partially attributed to individual personality traits and personal factors, such as being a parent or having completed vocational training prior to university, as well as one’s study program itself.

In our study, BSc Midwifery students reported the highest levels of empathy, followed by MSc Health and Nursing Sciences students, medical students, and finally BSc Nursing students. These results partially align with previous research, although international comparisons yield mixed patterns. For instance, medical students in our sample scored within the European average [47, 54] while BSc Nursing and MSc Health and Nursing Sciences students scored below most published international benchmarks [55–59]. Some studies have similarly found lower empathy levels among nurses and nursing students compared to physicians and midwifery students, though other studies report no significant group differences. However, interdisciplinary comparisons, especially those including midwives, remain scarce.

### Social dominance orientation

SDO emerged as a substantial factor negatively affecting empathy across the sample. This aligns with previous findings from studies in the general population linking SDO to reduced prosocial behavior, lower Agreeableness, and limited concern for others [28]. A preference for hierarchy may lead individuals to interpret relationships through the lens of power and status, prioritizing control over collaboration. In healthcare contexts, this risks undermining the relational competencies necessary for empathetic clinical care and interprofessional collaboration, ultimately compromising patient-centered care [60, 61].

These implications are particularly troubling given long-standing concerns about the role of hierarchy in medical training and clinical practice [62]: While an empathy decline in medical students is well documented [16–19] its causes remain debated. A Belgian study found that SDO tends to increase during medical training [63] suggesting that students who are constantly exposed to a strongly hierarchical work environment may internalize hierarchical norms over time. In this light, our findings, associating higher SDO with lower empathy, may thus help illuminate a key mechanism: students may not be simply failing to acquire empathy, but may be learning to deprioritize it in favor of dominance and control. Within hierarchical systems, students adapt to belong, observing and replicating the informal values embedded in clinical practice. This resonates with prior research on the hidden curriculum and the absence of positive role models, which have been shown to hinder empathy [16, 22, 27].

The rising SDO may also reflect processes of professionalization and boundary-drawing, students distancing themselves from patients, perhaps due to the false belief that emotional distancing provides emotional self-preservation [5, 16, 23] and from other professions as a way of asserting role-specific authority. In this light, empathy is not merely an individual trait but a social practice shaped by institutional logics that often reward detachment and dominance.

Interestingly, nursing students in our study reported the highest levels of SDO. Though counterintuitive, this may reflect their unique position within an unresolved professional transition. In Germany, nursing has only recently begun its shift from vocational training to academic education. While BSc-qualified nurses receive a higher level of training, this is not yet matched by formal differentiation in roles, responsibilities, or career progression once they take up work as nurses in the hospital. These students may be perceived as overstepping by vocationally trained peers while still receiving little recognition from physicians [64–66]. In this ambiguous and unrewarded position, heightened SDO may reflect a defensive response, a way to assert status, or a desire to establish professional distinction in a system that offers little structural validation.

Taken together, these findings reinforce calls for critical engagement with how hierarchy is faced in health professions education. Hierarchical structures not only shape educational experiences, but they also mold professional identities and interpersonal dynamics, and ultimately, patient outcomes. This is particularly relevant in healthcare systems like Germany’s, where interprofessional boundaries remain rigid, physicians retain legal and organizational authority, and non-physician roles are often structurally subordinate, dynamics that may reinforce hierarchical norms even when not explicitly taught or endorsed.

### Additional findings

While our primary focus was on the relationship between Social Dominance Orientation and empathy, our second research question addressed how individual factors may contribute to differences in empathy across student groups. In this regard, several secondary findings offer relevant context for interpreting variations in empathy across groups.

Empathy was positively associated with the personality traits Agreeableness and Openness, as well as with sociodemographic characteristics including being a parent, holding religious or spiritual beliefs, and higher subjective social status. These findings align with previous research suggesting that life experience, community affiliation, and sense of social stability can enhance empathy [28, 29]^67^– [71]. While these traits are mostly not directly modifiable, they may help identify students who could benefit from additional support or targeted interventions.

Differences between nursing and midwifery students, despite similar program structures and professional values, further highlight the influence of structural and institutional factors. Midwifery students received stipends and more consistent mentorship, while nursing students reported limited financial support, recognition, and career prospects. These factors likely contributed to higher levels of stress, and, therefore, reduced empathy, consistent with the Effort-Reward Imbalance model, which links high demands paired with inadequate rewards to emotional exhaustion and diminished empathy [26] 64–66, 72]. In this context, empathy depends not only on personal competence but also structural investment.

Lastly, the discrepancy between self-assessed and measured empathy scores adds nuance to our findings. Nursing and midwifery students tended to overestimate their empathy, possibly due to stronger social desirability bias in professions where empathy is central to identity [73]. In contrast, medical students slightly underestimated theirs, potentially reflecting a culture that prioritizes technical over emotional competence [16]. These patterns may also involve metacognitive bias, such as the Dunning-Kruger [74] effect, where less empathetic individuals are unaware of their limitations, and more empathetic ones are more self-critical.

These findings support the broader view that empathy in healthcare education is shaped by a complex interplay of individual, professional, and institutional factors.

### Practical implications

While some determinants of empathy, such as personality or sociodemographic background, may be relatively stable, or not amenable to direct intervention, both empathy and SDO are considered adaptable constructs [19, 21, 75]. In light of our findings, which suggest that higher SDO is associated with lower empathy, targeted efforts to address hierarchical attitudes may offer a meaningful opportunity to shape a more empathetic healthcare workforce.

Evidence supports the effectiveness of empathy-enhancing interventions [19, 21] many of which can be adapted to the interdisciplinary and hierarchical realities of healthcare. Educational formats such as Interprofessional Education (IPE) have been shown to reduce status-based barriers and promote mutual respect [76]. Likewise, narrative exercises and simulation-based training can deepen emotional insight and improve empathetic communication [77–79]. At Martin Luther University Halle-Wittenberg guided debriefings in midwifery education provide a structured and safe environment to process difficult encounters and reflect on underlying power dynamics. Introducing similar formats across other disciplines may not only enhance empathy but also challenge implicit hierarchies and encourage interprofessional dialogue.

We propose establishing regular, interdisciplinary small-group reflection sessions, moderated by trained facilitators, where students can share and process experiences related to empathy, power, and role expectations. In addition, a recurrent, anonymous reporting forum could allow students to submit incidents involving empathy lapses or hierarchical tensions during training. These anonymized cases could then serve as the basis for structured group discussions, offering a space for critical reflection without fear of judgment or exposure.

To sustain and institutionalize these efforts, medical schools should systematically review their curricula and learning environments for factors that may suppress empathy or reproduce dominance-based norms, such as insufficient opportunities and limited time for emotional processing, the absence of empathetic role models, or the presence of dehumanizing or biased educational content, and actively seek opportunities to encourage empathetic behavior and reduce power imbalances. Reform should not be confined to the classroom, but these changes must be supported by broader structural measures: equitable access to financial support, robust mentorship programs, and inclusive, collaborative clinical policies are essential for cultivating learning environments that reward empathy and cooperation rather than hierarchy and detachment.

### Limitations

The study relies entirely on self-assessment through questionnaires, making it susceptible to social desirability bias, especially on an issue as central to healthcare as empathy. Participants may have responded in ways that align with perceived professional norms rather than providing fully accurate self-evaluations. This is a known limitation of self-report tools and may contribute to the divergence observed between students’ subjective ratings and their scores on the JSE-HPS. To address this, future research could benefit from complementing empathy assessments with alternative measures, such as third-party observations, like peer or faculty evaluations, simulated patient feedback, or performance-based tools like Objective Structured Clinical Examinations (OSCEs). Additionally, the use of established social desirability instruments, such as the Marlowe-Crowne Social Desirability Scale [80] or the Balanced Inventory of Desirable Responding (BIDR) [81] could help account for response bias and strengthen construct validity.

Beyond that, our sample size is relatively small, despite a good response, and is limited to a single university in Germany. This institutional and geographic specificity may further limit the generalizability of our findings. At the same time, the cultural and structural characteristics of the German healthcare and education systems may influence both empathy and hierarchical attitudes among healthcare students. Future studies should obtain larger, multi-institutional samples to enhance generalizability.

While our findings reveal statistically robust negative associations between Social Dominance Orientation and empathy, they are based on cross-sectional data and therefore do not permit causal conclusions or determine the direction of effects. The observed patterns align with theoretical expectations, but it remains unclear whether higher SDO contributes to lower empathy, whether reduced empathy reinforces dominance-oriented attitudes, or whether both are influenced by other underlying factors. Nevertheless, identifying this association adds important empirical support to the growing discourse on empathy in healthcare education and clinical training. Future research using longitudinal or experimental designs could help clarify these dynamics, offer valuable insights into practical applications, and determine whether targeted interventions to foster empathy may also influence hierarchical attitudes – and vice versa.

## Conclusions

Overall, this study points to a broader complexity in how empathy is cultivated within healthcare education. The relationship between empathy and its determinants is complex and cannot easily be interpreted outside the context in which it is experienced, especially across disciplines shaped by differing social expectations and professional norms. Variations in empathy across student groups reflect its nature not only as a taught skill, but also as a performance, an internalized value, and as socially negotiated, shaped by personal experiences, personality traits, professional roles, and systemic conditions.

Our findings highlight the need to address hierarchical attitudes and professional norms as part of empathy development in healthcare education. Supporting empathy requires more than individual skill-building; it calls for institutional strategies that engage with power dynamics, role expectations, and structural inequalities. While individual-level interventions are valuable, sustainable change may depend on deeper cultural and systemic reform.

Creating spaces for critical reflection, such as narrative work, structured debriefings, or interprofessional discussions, may help students to safely explore their assumptions and values and develop a more authentic, resilient, and sustainable understanding of empathy in clinical practice. Beyond these pedagogical approaches, it may be necessary to question the hierarchical norms embedded in biomedical education and medical practice, and promote institutional policies that prioritize equity, collaboration, and emotional literacy in clinical environments.

Ultimately, fostering empathy in healthcare requires not only personal development but also a critical engagement with the professional norms and power structures that shape how empathy is learned, practiced, and valued.

## Electronic supplementary material

Below is the link to the electronic supplementary material.

Supplementary Material 1

Supplementary Material 2

## Data Availability

The datasets used and/or analyzed during the current study are available from the corresponding author on reasonable request.

## Abbreviations

BIDR: Balanced Inventory of Desirable Responding
CC: Compassionate Care
IPE: Interprofessional education
JSE-HPS: Jefferson Scale of Empathy for Health Profession Students
KSDO-3: Short Scale on Social Dominance Orientation
MSc HNS: MSc Health and Nursing Sciences
NEO-FFI-30: NEO-Five-Factor-Inventory
NPI-15: Narcissistic Personality Inventory
OSCE: Objective Structured Clinical Examinations
PT: Perspective Taking
SDO: Social Dominance Orientation
SA: Self-assessment
SSS: Subjective social status
WPS: Walking in Patients’ Shoes
