# Supplementary material for: Hierarchy hurts: a comparative cross-sectional analysis of empathy and its determinants in medical, midwifery, and nursing students
Source: BMC Med Educ. 2025 Jul 18;25:1076. doi: 10.1186/s12909-025-07683-w (PMC12273016; doi:10.1186/s12909-025-07683-w)
Supplement: Supplementary file 1 — Supplementary Material 1 [file 12909_2025_7683_MOESM1_ESM.docx]

Appendix 1: English-language version of the socio-demographic questionnaire

# Socio-demographic background

Now, we would like to ask you a few questions about your personal circumstances.

In which year were you born?

________________________

What is your gender identity?

________________________

Which German state did you grow up?

________________________

Did you grow up with siblings?

 Yes  No

If yes, what is your birth order?

 Eldest child  Middle child  Youngest child

Would you describe yourself as religious, spiritual, or believing?

 Yes  No

What is your current relationship status?

 Single

 In a relationship

 Married/ in a registered partnership

 Divorced/ registered partnership dissolved

 Widowed/ registered partner deceased

 Other: ____________

Do you have children?

 Yes  No

If yes, how many children do you have?

________________________

What is you major?

 Medicine

 Master’s in Health and Nursing Sciences

 Bachelor’s in Nursing

 Bachelor’s in Midwifery

 Other: ____________

What semester are you currently in?

________________________

What is your current GPA?

________________________

What grade did you receive in your Bachelor’s degree?

________________________

What grade did you receive in your first medical state examination?

________________________

Have you already completed any vocational training?

 Yes  No

If yes, in which profession did you complete your training?

________________________

Are you working while studying?

 Yes  No

If yes, how many hours do you work on average per week?

 less than 5 hours

 5 - 10 hours

 11 - 20 hours

 21 - 30 hours

 over 30 hours

What is your monthly available income before expenses such as rent, utilities, and groceries?

 less than 520€

 520€ - 1000€

 1001€ - 1600€

 1601€ - 2000€

 over 2000€

Which of the following best describes your mother’s profession?

(If you do not have a mother, please leave the field blank.)

- Member of a legislative body, senior public administration official, or executive in the private sector

 Member of a legislative body or senior public administration official

 Business executive or department head in large companies

 Head of a smaller company

- Scientist or doctor

 Physicist, mathematician, or engineer

 Biologist

 Doctor

 Scientific teacher

 Other scientist or related professions (e.g., lawyer, business consultant, clergyman, social scientist, etc.)

- Technician or similarly skilled non-technical profession (e.g., medical professional)

 Technical professional

 Life science or healthcare professional

 Non-scientific teacher

 Other specialist (e.g., tax specialist, police commissioner, entertainment or sports profession)

- Office worker or commercial employee

 Office worker without customer contact

 Office worker with customer contact

- Service profession (e.g., care worker) or sales in shops and markets

 Person-related service profession (e.g., care worker) or security worker

 Model, saleswoman, demonstrator

 Skilled worker in agriculture or fishing

- Craft or related profession

 Mining or construction profession

 Metalworker, mechanic, or related profession

 Precision worker, artisan, printer, or related profession

 Other craft or related profession (e.g., carpenter, textile profession, shoemaker)

- Equipment or machine operator or assembler

 Operator of stationary or related equipment

 Machine operator or assembler

 Vehicle driver or operator of mobile equipment

- Unskilled worker

 Sales or service worker

 Agricultural, fishing, or related unskilled worker

 Unskilled worker in mining, construction, manufacturing, or transportation

 Soldier

 Unemployed

 Other: ____________

If your mother is a doctor, what field is she working in?

 General medicine

 Anesthesiology

 Occupational medicine

 Ophthalmology

 Biochemistry

 General surgery

 Gynaecology and Obstetrics

 Otorhinolaryngology

 Dermatology

 Human genetics

 Hygiene and environmental medicine

 Internal medicine

 Paediatric and adolescent medicine

 Paediatric psychiatry and psychotherapy

 Laboratory medicine

 Microbiology, virology and infection epidemiology

 Oral and maxillofacial surgery

 Neurosurgery

 Neurology

 Nuclear medicine

 Public health

 Pathology

 Pharmacology

 Phoniatrics and paediatric audiology

 Physical and rehabilitative medicine

 Physiology

 Psychiatry and psychotherapy

 Psychosomatic medicine and psychotherapy

 Radiology

 Forensic medicine

 Radiation therapy

 Transfusion medicine

 Urology

Which of the following best describes your father’s profession?

(If you do not have a father, please leave the field blank.)

- Member of a legislative body, senior public administration official, or executive in the private sector

 Member of a legislative body or senior public administration official

 Business executive or department head in large companies

 Head of a smaller company

- Scientist or doctor

 Physicist, mathematician, or engineer

 Biologist

 Doctor

 Scientific teacher

 Other scientist or related professions (e.g., lawyer, business consultant, clergyman, social scientist, etc.)

- Technician or similarly skilled non-technical profession (e.g., medical professional)

 Technical professional

 Life science or healthcare professional

 Non-scientific teacher

 Other specialist (e.g., tax specialist, police commissioner, entertainment or sports profession)

- Office worker or commercial employee

 Office worker without customer contact

 Office worker with customer contact

- Service profession (e.g., care worker) or sales in shops and markets

 Person-related service profession (e.g., care worker) or security worker

 Model, saleswoman, demonstrator

 Skilled worker in agriculture or fishing

- Craft or related profession

 Mining or construction profession

 Metalworker, mechanic, or related profession

 Precision worker, artisan, printer, or related profession

 Other craft or related profession (e.g., carpenter, textile profession, shoemaker)

- Equipment or machine operator or assembler

 Operator of stationary or related equipment

 Machine operator or assembler

 Vehicle driver or operator of mobile equipment

- Unskilled worker

 Sales or service worker

 Agricultural, fishing, or related unskilled worker

 Unskilled worker in mining, construction, manufacturing, or transportation

 Soldier

 Unemployed

 Other: ____________

If your father is a doctor, what field is he working in?

 General medicine

 Anesthesiology

 Occupational medicine

 Ophthalmology

 Biochemistry

 Surgery

 Gynaecology and Obstetrics

 Otorhinolaryngology

 Dermatology

 Human genetics

 Hygiene and environmental medicine

 Internal medicine

 Paediatric and adolescent medicine

 Paediatric psychiatry and psychotherapy

 Laboratory medicine

 Microbiology, virology and infection epidemiology

 Oral and maxillofacial surgery

 Neurosurgery

 Neurology

 Nuclear medicine

 Public health

 Pathology

 Pharmacology

 Phoniatrics and paediatric audiology

 Physical and rehabilitative medicine

 Physiology

 Psychiatry and psychotherapy

 Psychosomatic medicine and psychotherapy

 Radiology

 Forensic medicine

 Radiation therapy

 Transfusion medicine

 Urology

# Experience as a patient

Finally, we would like to ask about your personal experiences with illness.

Have you ever been hospitalized as a patient?

 Yes, frequently/ regularly.

 Yes, multiple times.

 Yes, once.

 No, never.

Has an important person in your life ever been seriously ill?

 Yes  No
